# Live birth following IVF/ICSI using oocytes from donor who was conceived via IVF: a case report

**DOI:** 10.1007/s10815-015-0590-3

**Published:** 2015-10-08

**Authors:** Shahryar K. Kavoussi, Kate C. Odenwald, Roxanne B. Summers-Colquitt, Parviz K. Kavoussi, K. M. Kavoussi, Caitlin L. Shelinbarger, Thomas B. Pool

**Affiliations:** Austin Fertility & Reproductive Medicine/Westlake IVF, 300 Beardsley Lane, Bldg B, Suite 200, Austin, TX 78746 USA

**Keywords:** Donor oocytes, Oocyte donor, IVF, IVF/ICSI

## Abstract

**Purpose:**

The purpose of the study was to report a case of live birth following donor oocyte in vitro fertilization/intracytoplasmic sperm injection (IVF/ICSI) in which the oocyte donor herself was conceived via IVF. To our knowledge, such a case has not been previously reported.

**Methods:**

Retrospective chart review; this case is reported after chart review of a successful outcome.

**Results:**

A 42 year-old woman, with diminished ovarian reserve, and her husband desired to conceive. She underwent a fresh IVF/ICSI cycle with her own oocytes, which unfortunately was not fruitful in terms of pregnancy or cryopreserved embryos. The couple was counseled regarding the option of donor oocytes, and they elected to proceed with a fresh cycle of donor oocyte IVF/ICSI. The couple selected an anonymous oocyte donor from a donor agency who was a first-time oocyte donor and, interestingly, was conceived via IVF herself. The fresh donor oocyte/IVF/ICSI cycle did not result in pregnancy; however, two supernumerary blastocysts were cryopreserved for future cycles. The recipient’s subsequent frozen-thawed embryo transfer (FET) resulted in a singleton gestation and live birth.

**Conclusions:**

An oocyte donor who was conceived via IVF had good ovarian response to stimulation, a good number of oocytes retrieved, and the formation and cryopreservation of blastocysts which, in a subsequent FET cycle, resulted in pregnancy and live birth for a recipient couple. To our knowledge, this is the first case reported of live birth with the use of donor oocytes from an oocyte donor who herself was conceived via IVF.

## Introduction

During the initial years of human clinical in vitro fertilization (IVF) [1], it had been assumed that, in the absence of a significant genetic and/or metabolic condition that may impact reproductive capacity, female children born as a result of IVF would have good fertility potential when they were to reach reproductive age. Louise Brown, the first person to be born after having been conceived via IVF, gave birth to a child of her own in 2008 and again in 2013. In addition, Elizabeth Carr Comeau, the first person to be born in the US after IVF, conceived spontaneously and delivered her first child in 2010. Many other women who were conceived via IVF have delivered children of their own as well. The ovarian reserve of oocyte donors is a key component of their suitability for oocyte donation, and anti-müllerian hormone (AMH) has been a strong predictor of ovarian response to stimulation protocols [2]. We report a case that to our knowledge, is the first report of live birth following donor oocytes used for IVF/ICSI from an oocyte donor who herself was conceived via IVF.

## Materials and methods

This case is reported after chart review of a successful outcome. Patient-informed consent was not requested since all patient data was de-identified. A computerized search was performed in PubMed and Medline databases. The keywords and various combinations of phrases used in the search included the following: “IVF offspring”, “IVF children”, “female fertility”, “female infertility”, “own fertility”, “women born from IVF”, “conceive”, “fertility”, “oocyte donor”, “egg donor”, “ART offspring”, and “ART children”. None of the encountered abstracts had reported the findings of this case report.

## Results

A 42 year-old nulligravid woman, with diminished ovarian reserve, and her husband were interested in an IVF/ICSI cycle with donor oocytes after having undergone an autologous IVF/ICSI cycle with her own oocytes which resulted in a negative serum hCG level without surplus embryos for cryopreservation. They selected a first-time screened and tested 23 year-old anonymous oocyte donor who was available through an oocyte donation agency. The oocyte donor’s AMH level was 4.2 ng/ml. Interestingly, her medical history was significant for having been conceived via IVF. Her comments reflected having being raised by her parents to have a great appreciation for the IVF process and a desire to help women who cannot have children to experience the joy of doing so with assistance. She had previously delivered two children of her own which she had conceived without difficulty.

The oocyte donor’s ovarian stimulation protocol consisted of an antagonist protocol with 300 IU of gonadotropins per day. After 11 days of ovarian stimulation, the oocyte donor’s peak estradiol level was 1369 pg/ml and her progesterone level was 0.8 ng/ml. Recombinant hCG (Ovidrel) was administered subcutaneously for final oocyte maturation, and 27 oocytes were retrieved 35 h later. Nineteen oocytes were M2 oocytes. ICSI was performed for the 19 mature oocytes, of which 11 fertilized and yielded 2PN zygotes and two yielded 3PN embryos; six mature oocytes did not fertilize. Ten of the 11 two pronuclear zygotes underwent cleavage and on day 3, two embryos, each graded 8A in accordance with the SART embryo grading system, were used for embryo transfer to the recipient’s uterus. In addition, two day 6 surplus blastocysts, each graded 3BB in accordance with the Gardner embryo scoring system, were cryopreserved by means of vitrification. The recipient unfortunately had a negative pregnancy test as a result of her fresh embryo transfer.

The couple proceeded with frozen-thawed embryo transfer 4 months later. The wife’s endometrial preparation with an estradiol and progesterone protocol resulted in a trilaminar endometrium with a thickness of 9.0 mm. The two cryopreserved blastocysts were thawed and transferred to the patient’s uterus, resulting in pregnancy and live birth of a healthy singleton gestation.

## Discussion

Since the initial years of IVF, many women who were conceived via assisted reproductive technologies (ART) have reached reproductive age and have had children of their own. Parallel to this progression has been a significant increase in the utilization of donor oocytes in recent years [3]. Success rates for donor oocyte treatment cycles have been shown to be relatively stable for recipients who are into their mid-forties and beyond [4, 5]. This case report illustrates the case of an oocyte donor who was conceived via IVF and had a good response to controlled ovarian stimulation, a good number of oocytes retrieved, and the formation and cryopreservation of good quality supernumerary blastocysts which, in a subsequent FET cycle, resulted in pregnancy and live birth for a recipient couple. To our knowledge, this is the first reported case of live birth following IVF/ICSI using oocytes from an oocyte donor who was conceived via IVF herself. This further supports the data that women born from IVF not only have good fertility potential but also may meet criteria to be oocyte donors as well.

## References

[1] Brinsden PR, Brinsden PR (2009). Thirty years of IVF: the legacy of Patrick Steptoe and Robert Edwards. Hum Fertil (Camb).

[2] Nakhuda GS, Douglas NC, Thornton MH, Guarnaccia MM, Lobo R, Sauer MV (2010). Anti-müllerian hormone testing is useful for individualization of stimulation protocols in oocyte donors. Reprod Biomed Online.

[3] Kawwass JF, Monsour M, Crawford S, Kissin DM, Session DR, Kulkarni AD (2013). Jamieson DJ; National ART Surveillance System (NASS) group. Trends and outcomes for donor oocyte cycles in the United States, 2000-2010. JAMA.

[4] Yeh JS, Steward RG, Dude AM, Shah AA, Goldfarb JM, Muasher SJ (2014). Pregnancy rates in donor oocyte cycles compared to similar autologous in vitro fertilization cycles: an analysis of 26,457 fresh cycles from the Society for Assisted Reproductive Technology. Fertil Steril.

[5] Yeh JS, Steward RG, Dude AM, Shah AA, Goldfarb JM, Muasher SJ (2014). Pregnancy outcomes decline in recipients over age 44: an analysis of 27,959 fresh donor oocyte in vitro fertilization cycles from the Society for Assisted Reproductive Technology. Fertil Steril.

